# Spatial Pattern and Environmental Drivers of Acid Phosphatase Activity in Europe

**DOI:** 10.3389/fdata.2019.00051

**Published:** 2020-01-23

**Authors:** Yan Sun, Daniel S. Goll, Philippe Ciais, Shushi Peng, Olga Margalef, Dolores Asensio, Jordi Sardans, Josep Peñuelas

**Affiliations:** ^1^Laboratoire des Sciences du Climat et de 1'Environnement, CEA-CNRS-UVSQ, Gif sur Yvette, France; ^2^Institute of Geography, University of Augsburg, Augsburg, Germany; ^3^Sino-French Institute for Earth System Science, College of Urban and Environmental Sciences, Peking University, Beijing, China; ^4^CSIC, Global Ecology Unit, Centre de Recerca Ecològica i Aplicacions Forestals, Consejo Superior de Investigaciones Científicas, UAB, Bellaterra, Spain; ^5^Centre de Recerca Ecològica i Aplicacions Forestals, Cerdanyola del Vallès, Spain

**Keywords:** back-propagation artificial network, Europe, phosphorus cycling, partial correlation analysis, regression tree, soil acid phosphatase

## Abstract

Acid phosphatase produced by plants and microbes plays a fundamental role in the recycling of soil phosphorus (P). A quantification of the spatial variation in potential acid phosphatase activity (AP) on large spatial scales and its drivers can help to reduce the uncertainty in our understanding of bio-availability of soil P. We applied two machine-learning methods (Random forests and back-propagation artificial networks) to simulate the spatial patterns of AP across Europe by scaling up 126 site observations of potential AP activity from field samples measured in the laboratory, using 12 environmental drivers as predictors. The back-propagation artificial network (BPN) method explained 58% of AP variability, more than the regression tree model (49%). In addition, BPN was able to identify the gradients in AP along three transects in Europe. Partial correlation analysis revealed that soil nutrients (total nitrogen, total P, and labile organic P) and climatic controls (annual precipitation, mean annual temperature, and temperature amplitude) were the dominant factors influencing AP variations in space. Higher AP occurred in regions with higher mean annual temperature, precipitation and higher soil total nitrogen. Soil TP and Po were non-monotonically correlated with modeled AP for Europe, indicating diffident strategies of P utilization by biomes in arid and humid area. This study helps to separate the influences of each factor on AP production and to reduce the uncertainty in estimating soil P availability. The BPN model trained with European data, however, could not produce a robust global map of AP due to the lack of representative measurements of AP for tropical regions. Filling this data gap will help us to understand the physiological basis of P-use strategies in natural soils.

## Introduction

Phosphorus (P) is an essential nutrient for all living organisms (White and Hammond, 2008). As weathering of minerals and the deposition of atmospheric dust are minor sources of P (Walker and Syers, 1976; Vitousek et al., 2010; Wang et al., 2015), the recycling of organic P from litter and soil organic matter is of utter importance for plant growth and microbial activity in terrestrial ecosystems. In P-poor ecosystems, limited P recycling may dampen the response of plant growth to elevated CO_2_ concentration (Ellsworth et al., 2017). Yang et al. (2014) reported in a modeling study that the effect of elevated CO_2_ on plant productivity in the Amazon Basin critically dependent on assumptions regarding the P-recycling efficiency within soils, which was strongly related to the parameterization of phosphatase production in their model.

The rate at which ecosystems can recycle P from litter and soil organic matter is poorly quantified by observation (Gill and Finzi, 2016). Soil phosphatases secreted by fungi, bacteria, and plant roots play an important but poorly quantified role in transforming complex and unavailable forms of organic P into assimilable phosphate (Caldwell, 2005). Potential phosphatase activity in soils, which can be measured in the lab from soil samples, is an indicator of the capacity of enzyme communities to cleave organic molecules containing P (Krämer and Green, 2000), and serves as a surrogate for the lacking measurements of P mineralization in the soil.

Potential phosphatase activity measured in the laboratory under optimal conditions (optimal temperature, well-mixed soil, no water limitation; Eivazi and Tabatabai, 1977) provides an upper limit of their actual activity in a soil (Nannipieri et al., 2011; Margalef et al., 2017), which cannot be directly measured. Acid phosphatases (AP) are more widespread than alkaline phosphatases at soil pH values representative of most natural soils (Margalef et al., 2017), which justifies our focus on AP in this study.

Many experiments have investigated the responses of potential AP activity to fertilization (Marklein and Houlton, 2012; Maistry et al., 2015), temperature changes (Sardans et al., 2006), and water availability (Sardans and Peñuelas, 2005; Sardans et al., 2006; Zhou et al., 2013) or to other disturbances under controlled conditions (Sinsabaugh et al., 2008). Gradients of AP along transects have been measured in few regions (Brockett et al., 2012; Huang et al., 2013; Kitayama, 2013). They found that warming, increasing soil water and nitrogen availability can enhance AP activity. However, these studies were limited to site or small region scale and only considering a subset of potential environmental factors. Recently, Margalef et al. (2017) addressed this gap, by compiling a global data set of phosphatase activity and using correlation analysis, regression analysis and structural equation models (SEMs) to provide insights of the drivers of phosphatase activity distribution on global scale. However, the approach used by Margalef et al. (2017) cannot account for non-linear responses to different variables (Ma et al., 2017) and omitted important variable like soil labile P.

The benchmarking of the growing number of land models which include phosphorus cycling is currently hampered by the lack of spatial explicit information on AP on regional to global scale. The limited understanding of the drivers responsible for differences in AP across different ecosystems and climatic and soils conditions further hampers the global efforts of including P cycles in the land surface models (Reed et al., 2015).

Machine learning (ML) is a family of approaches which has been increasingly used to identify patterns in complex ecological data sets and scale up site measurements (e.g., Papale and Valentini, 2003; Golubiewski, 2006; Wiesmeier et al., 2011; Keenan et al., 2012; Were et al., 2015), but have not been used for upscaling the spatial patterns of AP.

We used two ML methods in combination with gridded fields of environmental factors to upscale site data of potential AP (Margalef et al., 2017) to gridded AP fields for continental Europe. Then we identify the main drivers behind the spatial variation of upscaled AP in Europe. Finally, we used the best ML model trained by European data in a first attempt to produce a global map of AP, and this map is cross-validated using non-European data.

## Methods

**Figure 1:**
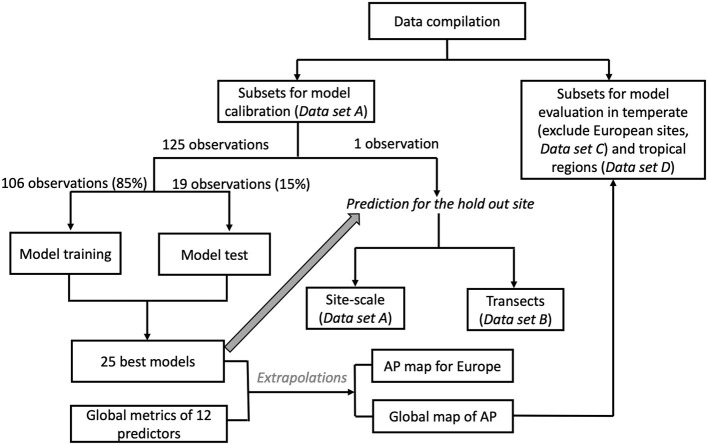
Flow chart for the calibration and evaluation of the prediction models.

AP measurements compiled by Margalef et al. (2017) are combined with gridded fields of 12 environmental factors to model spatial pattern of annual AP across Europe and the globe on a spatial resolution of 10 km. This was done by using back-propagation artificial network (BPNs) and Regression Trees (RT). Figure 1 shows the main procedure of model calibration and evaluation.

### Datasets

We used 296 measurements of AP from 139 published studies (Margalef et al., 2017). This dataset contained 54 tropical sites and 242 ex-tropical sites (155 in Europe). Location, soil pH and soil nutrient contents (total soil C, soil organic C, total nitrogen, and total P) for each site were obtained from the original publications.

We separated the data set into four subsets as shown in Figure 1: (1) data used to train the ML models (*Data set A*), (2) data used for cross-validating the spatial gradients of AP modeled for Europe (*Data set B*), (3) data used to evaluate AP patterns in temperate regions outside Europe (*Data set C*), and (4) data used to evaluate AP patterns in tropical regions (*Data set D*). *Dataset A* contains 126 European sites with complete information for the all the predictors listed below. *Data set B* contains all 155 European sites. *Data set C* and *Data set D* contained 87 temperate sites outside Europe and only 54 tropical sites, respectively.

We selected 12 variables as predictors for the upscaling based on findings from manipulation experiments (Hogan et al., 2010; Huang et al., 2011; Marklein and Houlton, 2012), observation time series (Sardans et al., 2006) and previous regression analyses (Margalef et al., 2017): soil pH (SoilpH), soil clay content (Clay), soil organic carbon (OC), soil total nitrogen (TN), soil total phosphorus (TP) and soil labile organic phosphorus (Po), net primary productivity (NPP), mean annual temperature (MAT), amplitude of yearly temperature (AMP), mean annual precipitation (MAP), soil type (SoilType), and vegetation type (VegType).

We extracted predictors from each original publication when available. In case information on predictors were not reported, we extracted the missing data from gridded datasets (Table 1) based on the geographical coordinates of each measurement site (Shangguan et al., 2014; Ballabio et al., 2016, 2019; Fick and Hijmans, 2017; Hengl et al., 2017; MOD12Q2 and MOD17). The databases for each predictor variables are listed in Table 1. Detailed information about the databases are provided in Supporting Information Appendix S1. Details on the gap filling of predictors can be found in Supporting Information Appendix S2. AP, NPP, AMP, and MAP, which followed a lognormal distribution, were log-transformed before build the prediction model (Supporting Information Appendix S2, Figure S2).

**Table 1:** Predictors used in prediction models and its source.

**Predictor name**	**Abbreviation**	**Source**	**Spatial resolution**	**References**
Soil organic carbon	OC	Soilgrids	250 m	Hengl et al., 2017
Soil pH	SoilpH	LUCAS	500 m	Ballabio et al., 2019
Soil total nitrogen	TN			
Soil total phosphorus	TP			
Soil clay content	Clay			Ballabio et al., 2016
Soil labile organic phosphorus	Po	Global maps of the soil P contents for different P forms TP from LUCAS USDA soil types and ratio of labile inorganic P and labile organic P	1 km	Yang et al., 2013; Hengl et al., 2017; Sun et al., 2017; Ballabio et al., 2019
Net primary productivity	NPP	MODIS-NPP (MOD17A3; mean value during 2000–2014)	1 km	Running et al., 2004; Zhao et al., 2005; Turner et al., 2006
Mean annual temperature	MAT	WorldClim	1 km	Fick and Hijmans, 2017
Yearly temperature amplitude	AMP			
Mean annual precipitation	MAP			
Soil type (categorical variable)	SoilType	Soilgrids and USDA class	250 m	Hengl et al., 2017
Vegetation type (categorical variable)	VegType	MODIS	1 km	Friedl et al., 2010

### Back-Propagation Artificial Networks

Back-propagation training algorithm (Kelley, 1960) is the most frequently used neural-network method (Were et al., 2015). These algorithms train networks until some targeted minimal error is achieved between the predicted and observed outputs (Kelley, 1960). We applied a BPN constituting a four-layer neural network: one input layer, two hidden layers and one output layer. Two categorical variables (vegetation type and soil type) were converted into vector form as BPN are not designed to handle categorical values following Mason et al. (2017). For example, the soil type has eight possible categories: (1) Entisol, (2) Inceptisol, (3) Aridsol, (4) Mollisol, (5) Alfisol, (6) Spodosol, (7) Ultisol, (8) Oxisol. An Entisol would have a vector value of [1 0 0 0 0 0 0 0], whereas a Alfisol would have a vector value of [0 0 0 0 1 0 0 0]. The input layer contained 34 nodes (10 numeral predictors and two categorical predictors; Table 1). The numbers of neurons for the two hidden layers were 10 and 5 for building the BPN model (Supporting Information Appendix S3, Figure S3). Each layer of the BPN was linked to prior and forward layers by weights that were determined using a gradient-descent learning algorithm, such as Widrow-Hoff learning rule (Kelley, 1960). We used the resilient back-propagation algorithm to update weights and biases along the negative of the gradient of the performance function (Riedmiller and Braun, 1993).

Each BPN runs multiple epochs until either of three convergence criteria is satisfied: (1) the number of training epochs reach up to 500, (2) the predicting bias is lower than 0.2, or (3) the number of validation checks failure reached 100 times (i.e., the predicting bias for validation sets doesn't decrease for 100 times). Criterion (1) and (2) helps to avoid the over-fitting issue. Criterion (3) helps to avoid additional simulations without any improvement of BPN model.

### Regression Trees

BPN models cannot rate the importance of predictors for accounting for differences in observed AP, so we also used RTs to build regression models. The input predictors, training and testing subset selection, leave-one-out validation and best model selection for the RT were the same as those used in the BPN method. The response data at each binary split were grouped into two descendant nodes to maximize homogeneity, and the best binary split was selected. The trees were grown to their maximum sizes following the tree template. We used an RT tree template where the minimal number of parents was 6, and the minimal number of observations at the terminal nodes of the trees was 3.

### Leave-One-Out Validation Method and BPN and RT Model Selection

The leave-one-out (LOO) cross validation method is best suited for model calibration and evaluation when observational data are scarce (Allen, 1974). LOO can test the reproducibility of prediction models using independent data (Cawley, 2006). Out of the 126 European sites (*Data set A*), 125 observations were used to train and select models and the remaining observation to verify the model. This procedure was repeated 126 times by excluding each observation once.

Within the BPN and RT models training and selection, the 126 observations were randomly separated into training (106 sites, 85%) and testing (19 sites, 15%) data sets (Figure 1). The BPN models were trained 5,000 times using different training subsets, and the RT models were trained 500 times. We used *R*^2^ and RMSE to assess the performance of the BPN and RT models on the training and testing data sets for each train-test possibility. We removed BPN and RT models with training *R*^2^ <0.6, testing *R*^2^ <0.5, training RMSE >15 μmol g^−1^ h^−1^ and testing RMSE >10 μmol g^−1^ h^−1^. Then, we selected the 25 models with the highest test-R^2^ for observed AP < 25 μmol g^−1^ h^−1^ and lowest RMSE for observed AP >25 μmol g^−1^ h^−1^. This selection algorithm ensured that the BNP and RT can accurately reproduce AP in both the training and testing data sets.

### Extrapolation of AP for Europe

**Figure 2:**
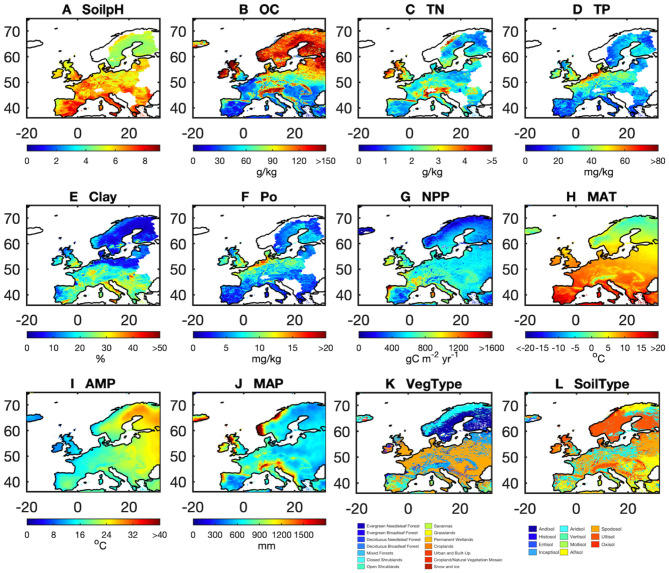
Spatial pattern of the 12 predictors: **(A)** soil pH (soilpH), **(B)** soil organic carbon content (OC), **(C)** soil total nitrogen content (TN), **(D)** soil total phosphorus content (TP), **(E)** clay content (Clay), **(F)** soil labile organic phosphorus content (Po), **(G)** net primary productivity (NPP), **(H)** mean annual temperature (MAT), **(I)** amplitude of yearly temperature (AMP), **(J)** mean annual precipitation (MAP), **(K)** vegetation cover (VegType), and **(L)** soil type (SoilType).

We resampled all of those gridded datasets of predictors (Table 1, Table S1) on a 10 km resolution using area-weighted mean methods for numerical predictors, and by using mode resampling for categorical predictors (i.e., vegetation types and soil types). Then we used the 25 selected model ensembles (25 best models × 126 LOO rounds) that produced AP with the highest correlation with observed AP in combination with gridded maps of the predictors at a resolution of 10 km (Figure 2) to scale up point observations to continental Europe (36–75°N and 20°W to 32°E). We first used the median of the 25 estimates for each LOO round and then derived the median estimates of AP across 126 LOO rounds to define the AP maps and their uncertainties across the European continent.

### Attribution of AP Pattern to Drivers Across Europe

ML models like BPN are able to extract hidden, complex, and generally subtle, non-linear relationships from scattered data and multiple predictors and use these relationships for up-scaling. The up-scaled AP maps are necessary but difficult to be interpreted by ML models due to the numbers of predictors and their interactions. We thus applied partial correlation analysis, a simple diagnostic analysis, on modeled maps of AP to gain a first order understanding of the most important predictors emerging from the model results in different regions.

The spatial partial correlation analysis for Europe was conducted at spatial resolution of half degree (~50 km). To do so, predictors were firstly resampled into half degree by using the same methods as described above. Then we performed the partial correlation analysis between AP and predictors (only numerical predictors) using a spatial moving window of 4.5 × 4.5°. This analysis partly avoided the collinearity among the predictors but still failed in separating effects of strongly collinear factors (e.g., NPP and climatic variables). Therefore, we performed (1) a partial correlation analysis between AP and all abiotic factors, and (2) a partial correlation analysis between AP, soil conditions and NPP without climatic variables. The median estimate of AP and the predictor metrics for Europe were first resampled to 0.5°. We thus selected 81 pixels surrounding each 0.5 grid cell for calculating the coefficients of partial correlation for AP and the predictors.

## Results

### Model Performance Across European Sites and Along Spatial Gradients

**Figure 3:**
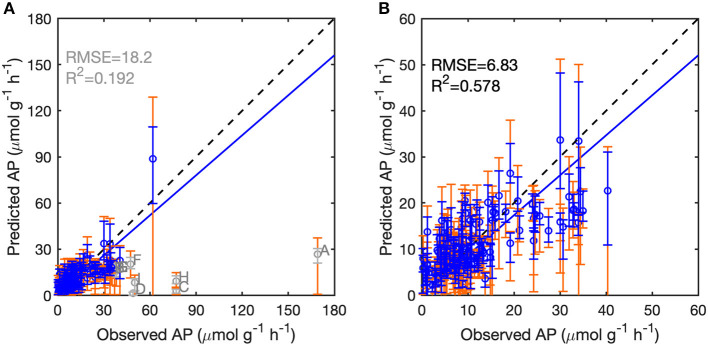
Performance of the BPN models on 126 European sites following the leave-one-out framework. **(A)** Predicted vs. observed acid phosphatase activity (AP) for all sites. **(B)** Predicted vs. observed AP with excluding seven outliers (gray circles). The dashed lines indicate the 1:1 lines. The blue lines indicate the regression lines between predicted and observed AP. The blue and gray error bars indicate the 25 and 75% quantiles of predicted AP, and the orange error bars indicate the 10 and 90% quantiles of predicted AP. The nine outliers are marked in gray: *Site A* and *D* are located in central England, *Site B* is in Northern Italy, *Site C* in Eastern Spain, *Site E* in Poland, *Site* F in Norway, and *Site G* in Western Spain.

We assessed the performance of the BPN and RT models for each of the 126 European sites (*Data set A*) by LOO cross-validation. Excluding nine outliers with absolute bias >20 μmol g^−1^ h^−1^ and relative bias >50% (Supporting Information Appendix S4, Figures S4, S5), the BPN models explained 58% of AP variability across all sites, with an RMSE of 6.83 μmol g^−1^ h^−1^ (Figure 3, Table S2). The BPN models reproduced 93% of the variance of AP for the remaining 117 sites. The observations of AP for 68 sites were within the uncertainty quantile range of 10–90% of AP simulated with the ensemble of 25 BPN models, and 32 site observations were within the 25–75% quantile range (Figure 3). The BPN model generally underestimated AP, but overestimated AP at sites with low activity (<20 μmol g^−1^ h^−1^), while AP at sites with intermediate and high observed activities (>20 μmol g^−1^ h^−1^) tended to be systematically underestimated. The BPN models outperformed the RT models for LOO validation (Table S2). With excluding 13 outliers, RT models explained 48.7% of the spatial variance of AP over all measurements, accompanied by a higher RMSE of 7.85 μmol g^−1^ h^−1^ (Figure S6, Table S2).

**Figure 4:**
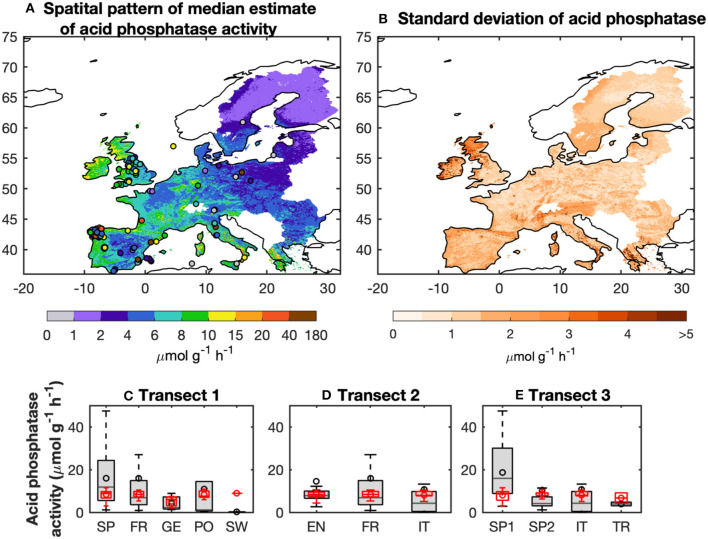
Spatial pattern of median estimates of acid phosphatase activity (AP) from the BPN models for Europe **(A)**. The observed AP are also shown. **(B)** Uncertainties of AP defined as the difference between the 75 and 25% quantiles of predicted AP from the BPN models. **(C–E)** show the changes of observed and predicted AP along three transects: **(C)** Spain (SP), France (FR), Germany (GE), Poland (PO), and Sweden (SW); **(D)** England (EN), France (FR), and Italy (IT); and **(E)** Western Spain (west of 5°W; SP1), Eastern Spain (east of 5°W; SP2), Italy (IT), and Turkey (TR).

Three transects (*Data set B*) were selected to determine if the BPN model could reproduce large scale observed spatial gradients (Figures 4C–E). *Transect 1* spanned across Spain, France, Germany, Poland and Sweden. Median estimates of modeled AP along this transect gradually decrease from 8.5 μmol g^−1^ h^−1^ in Spain to 5.6 μmol g^−1^ h^−1^ for Germany, which is roughly consistent with the observations (decrease from 11.8 to 2.3 μmol g^−1^ h^−1^). Modeled AP for sites in Poland and Sweden were all higher than observed values, though. *Transect 2* (across England and France to Italy) shows observed values going from 7.8 μmol g^−1^ h^−1^ (6.6–9.9 μmol g^−1^ h^−1^) in England to 4.2 μmol g^−1^ h^−1^ (0.4–9.8 μmol g^−1^ h^−1^) in Italy. Predicted AP along *Transect 2* give a smaller decrease than observed, going from 7.4 μmol g^−1^ h^−1^ (6.1–9.3 μmol g^−1^ h^−1^) to 7.2 μmol g^−1^ h^−1^ (5.5–8.8 μmol g^−1^ h^−1^). Modeled AP along *Transect 3* (from Western and Eastern Spain, Italy and Turkey) decreased from 8.5 μmol g^−1^ h^−1^ (5.1–10.8 μmol g^−1^ h^−1^) for Western Spain to 5.4 μmol g^−1^ h^−1^ for Turkey, a smaller decrease than the observations. This bias could be mainly attributed to the underestimation of modeled AP for sites with high values in Spain.

In summary, The BPN models are found in reproduce the spatial variance of AP for Europe and the direction of the AP gradients across ecological zones and climate, despite negative or positive biases compared to individual site-scale data points. Median BPN modeled estimate of AP across Europe increases from north to south, and also from east to west (Figure 4A). AP was highest for Northern England and Western Spain (10–40 μmol g^−1^ h^−1^), and lowest for Northern and Eastern Europe (<2 μmol g^−1^ h^−1^) (Figure 4). The uncertainty in the AP estimates (the difference between 75 and 25% quantiles; Figure S7) was low for most of Europe (<3 μmol g^−1^ h^−1^) except for England and Northern Europe (>3 μmol g^−1^ h^−1^).

### Relationship Between Modeled AP and Climate and Soil Conditions

**Figure 5:**
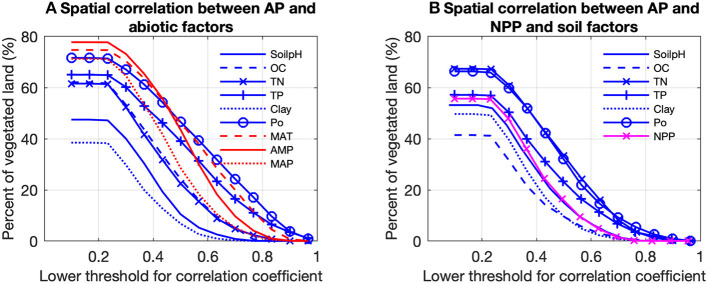
Proportions of vegetated land in Europe controlled by **(A)** climatic (red) and soil correlation analysis between predicted acid phosphatase activity (AP) and all numerical abiotic factors. **(B)** Was based on the partial correlation analysis between AP and NPP and soil factors. Pixels with *p* > 0.1 are excluded. The soil factors are: soil pH (SoilpH), soil organic carbon content (OC), soil total nitrogen content (TN), soil total phosphorus content (TP), soil clay content (Clay), soil labile organic phosphorus content (Po). The climatic factors are: mean annual temperature (MAT), amplitude of yearly temperature (AMP), and mean annual precipitation (MAP). For example, AP for about 48% of the vegetated land is controlled by soil pH, with partial correlation coefficients between AP and soil pH > 0.2.

**Figure 6:**
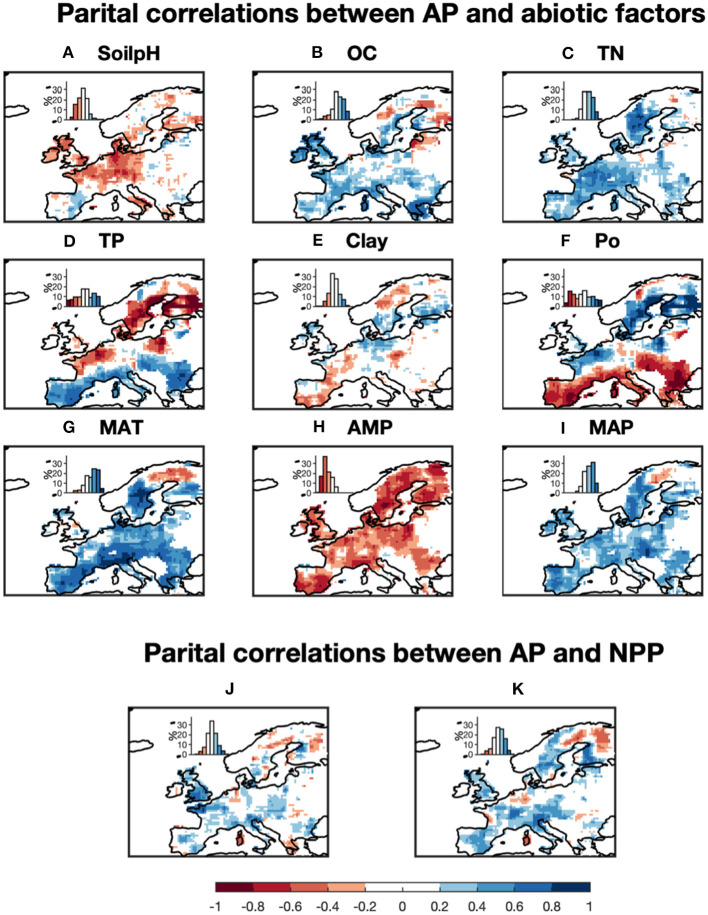
Partial correlations between the median estimates of acid phosphatase activity (AP) and 9 abiotic factors: **(A)** soil pH, **(B)** soil organic carbon, **(C)** soil total nitrogen content, **(D)** soil total phosphorus content, **(E)** soil clay content, **(F)** soil labile organic phosphorus content, **(G)** mean annual temperature, **(H)** amplitude of yearly temperature, and **(I)** mean annual precipitation. **(J,K)** Are the partial correlations between AP and NPP when including climatic variables and excluding climatic variables. Pixels with *p* > 0.1 were excluded. Frequency histograms showing the areal coverages correspond to positive and negative correlations, estimated as proportions of the total study area, are provided for each map (insets).

The partial correlation analysis indicated that correlations between gridded TP and Po and gridded climatic factors (MAT, AMP, and MAP) with gridded BPN estimates of AP for Europe were stronger than with the other factors (Figure 5). The proportions of land areas with significant (*p* < 0.1) correlations between AP and MAT, AMP and MAP were 76, 79, and 71%, respectively. AP was generally correlated with both MAT and MAP (Figures 6G–I), being positively associated with spatially increasing MAT, and negatively correlated with AMP.

When excluding climatic variables from partial correlation analysis, NPP has a significant correlation (*p* < 0.1) with AP for 58% of land areas. Significant positive correlations between NPP and AP are mainly found in Southern and Western Europe, while significant negative correlations are in Sweden and Finland (Figure 6K). The emerging relationship between modeled AP and NPP is consistent with those found between AP and climatic variables (Figures 6G–I,K) which reflects strong collinearity between NPP and climatic variables (Figure S9).

Modeled AP is found to be negatively correlated with soil pH at high soil pH values (6–9), which is common for Europe, especially in the Southern and the Western of the domain (Figure 6A). Soil nutrient contents also partly accounted for the spatial patterns of modeled AP. AP is found to be positively correlated with soil organic C content (OC) (Figures 6B–D,F). More specifically, AP and OC are significantly positively correlated (*p* < 0.1) in Southern and Western Europe but not significantly negatively correlated for Northern and central Europe. AP and TN are strongly positively correlated with AP in Southern and the Western Europe. AP is significantly (*p* < 0.1) positively correlated with TP but negatively correlated with Po in aridity area (P/PET < 0.7) (Figure 6; Figure S11), while AP is significantly (*p* < 0.1) negatively correlated with TP but positively correlated with Po in wetter region (P/PET > 1; Figure 6; Figure S11).

Modeled AP is found to be higher in highly weathered soils (Ultisols and Oxisols, 7.8–8.3 μmol g^−1^ h^−1^) compared to slightly and moderately weathered soils (1.2–7.2 μmol g^−1^ h^−1^) (Figure S8A). AP was the highest (8.9 μmol g^−1^ h^−1^) for Evergreen Broadleaf Forests and the lowest for Evergreen Needleleaf Forests among all of the biomes (Figure S8B).

### Upscaling of Site Data to a Global Map of AP

**Figure 7:**
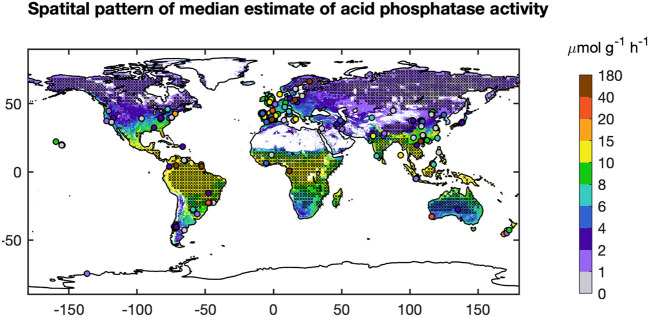
Global pattern of AP from the BPN models (median). “x” indicated area with mean annual temperature (MAT) <5°C or MAT > 20°C which falling out of the ranges of BPN built based on Europe sites. The observed AP are shown as dots.

We used the BPN models trained on European data in a first attempt to scale up AP to the global scale. The modeled global spatial pattern of AP show a clear latitudinal gradient of increasing AP from high latitudes to the equator (Figure 7). Modeled AP is also much higher for coastal than inland areas. The global up-scaled distribution of AP is however more uncertain than the European distribution. The BPN models explained only a small fraction (<15%) of AP (Supporting Information Appendix S5) variance over all measurements for both temperate sites outsides Europe (*Data set C*) and tropical regions (*Data set D*) (Figures S12A–D). The low prediction capacity for these sites may be partly attributed to the low accuracy of extracted gridded TN from ISRIC-WISE (Supporting Information Appendix S1). Excluding the sites with gap-filled TN, the BPN models explained 34% of AP variability for the rest of temperate sites outside Europe (Figures S12B,C). Besides, MAT of many sites fall out of the range of the Europe training sites, which also lead to the poor performance of our BNP models on sites outside Europe. Excluding sites with gap-filled TN and sites with MAT falling out the ranges between 5 and 20°C, the BPN models explained 42% of AP variability for temperate sites outside Europe within the MAT range of the training sites (5°C < MAT <20°C) (Figure 3). This indicates that the BPN can predict spatial gradients of AP with a reasonable accuracy (*R*^2^ = 0.58) over the European continent where the training data originate from, and still has some predictive ability at cross validation sites that originate from other temperate regions, mainly USA and China (Figure 7).

## Discussion

### Extrapolation of AP From Site to Regional and Global Scales

**Figure 8:**
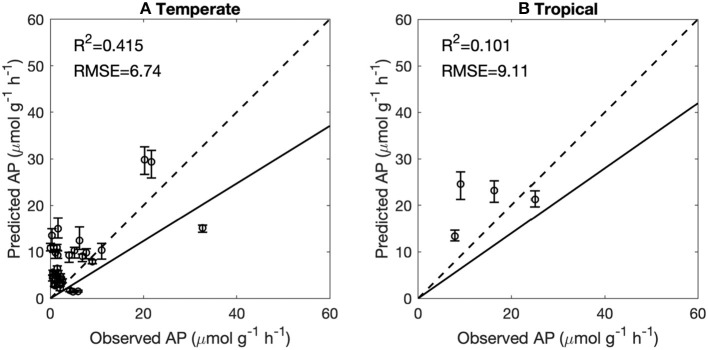
Performance of the back-propagation networks on temperate sites (excluding Europe) **(A)** and tropical sites **(B)**. Temperate and tropical sites with original TN and 5°C < MAT <20°C were reproduced using BPNs and were then compared to observations. The solid lines indicate the regression lines between predicted vs. observed AP. The dashed lines indicate the 1:1 lines. The error bars indicate the 10 and 90% quantiles of predicted AP.

This study is a first attempt to extrapolate AP site measurements from Europe to regional and global scales using ML methods. The BPN method successfully reproduced the spatial variance of AP across Europe. The performance and reproducibility of BPN models strongly depend on the quantity, quality, and representativeness of the training data set. We attribute the failure in extrapolation to global scale (Figure 8) to an insufficient number of representative training AP measurements, especially for tropical soils and climate.

AP activity in tropical regions varies widely from 0 to 80 μmol g^−1^ h^−1^. The BPN model trained across Europe was not able to capture this large range. A low representativeness of observations in Europe for vegetation types and climatic conditions outside Europe can partly account for this low prediction accuracy. The more homogenous climatic conditions in the tropics than the extra-tropics may increase the importance of soil conditions in accounting for AP variation (Supporting Information Appendix S6). Soil properties in the tropics are very heterogeneous and were shown to drive large scale patterns of ecosystem properties (Quesada et al., 2012). More AP measurements in tropical regions would be needed to help us understand the differences between soil types and P-use strategies of ecosystems in this region.

### Drivers of the Spatial Patterns of AP in Europe

The assessment of the major factors influencing potential AP help us to understand (1) the controls on AP across Europe and (2) the failure of our model to extrapolate to a global scale. Simple regression analyses were applied in previous studies to investigate the factors influencing AP (Sinsabaugh et al., 2008; Huang et al., 2013; Margalef et al., 2017). Such approaches, however, cannot manage collinearity among predictors nor account for non-linear responses to different variables (Ma et al., 2017). We applied partial correlation diagnostic to the output of the BPN model, which partly overcomes these shortcomings in separating the effect of each predictor on AP for different climatic zones and states of soil nutrients.

#### Relationships Between Modeled AP and Climatic Variables

Modeled AP is found to be positively correlated with MAT and MAP for most of Europe (Figures 6G,I) in line with results from manipulation experiments (soil warming and irrigation). Warming had a positive effect on AP in experimental studies for both temperate grassland soil (0–10 cm depth; Zhou et al., 2013) and Mediterranean shrubland (Sardans et al., 2006). A possible mechanism is that increasing temperature can accelerate plant growth and P acquisition by plants, thus reducing soil P content and stimulating AP production (Sardans et al., 2006). The depletion of soil P can stimulate the production of AP by both plants and soil microbes (Clarholm, 1993; Olander and Vitousek, 2000). Drought experiments in the Mediterranean reported no response (Sardans and Peñuelas, 2005) or reduction in AP activity (Sardans et al., 2006) under conditions of low soil-water content, associated with low water availability and plant growth and ultimately with a reduction in the P demand of plants and microbes. Zhou et al. (2013) reported that increased precipitation significantly increased soil AP in a temperate grassland at depths of 10–20 cm. Measurements based on spatial sampling have also indicated that a higher availability of soil water leads to a larger microbial biomass (Brockett et al., 2012), and hence increased activities of soil extracellular enzymes. Increasing precipitation should also promote substrate diffusion, which brings more soil NO_3_-N and NH_4_-N to the subsurface and increases AP (Zhou et al., 2013).

#### Relationships Between Modeled AP and Soil Variables

Nutrient availability, pH, organic C, and clay content are also found to play important roles in the modeled spatial variance of AP from west to east Europe (Figures 6A–F). Modeled AP was positively correlated with SOC. The substrate for AP, soil organic P, is tightly linked to SOC (Tipping et al., 2016), but not included in our analysis due to lack of data. Therefore, the correlation between AP and SOC can be interpreted as a control of substrate on AP. This finding was consistent with results from a meta-analysis of soil phosphatase activity and transect measurements (Sinsabaugh et al., 2008; Huang et al., 2013).

TN was also positively correlated with modeled AP for most of Europe (Figure 6C). This finding is consistent with results by Margalef et al. (2017) and a meta-analysis of N-fertilization experiments by Marklein and Houlton (2012). Higher soil N availability is supposed to strongly increase AP activity in both plant roots and bulk soils across a wide range of ecosystems (Marklein and Houlton, 2012).

In contrast to the monotonic behavior of AP with TN and climatic variables, soil TP and Po were non-monotonically correlated with modeled AP for Europe (Figures 6D,F). AP in wet regions (P/PET > 0.7) was negatively correlated with TP but positively correlated with Po. This could indicate that biological mineralization of Po via AP is an essential strategy to maintain the bioavailable P pool under humid conditions (Izquierdo et al., 2013). AP in aridity area (P/PET < 0.7) is positively correlated with TP but negatively correlated with Po (Figure 6). Compared with humid soils, arid soils typically contain lower SOM, thus the importance of minerals as source of P for biota is suggested to become more important (Lajtha and Schlesinger, 1988; Cross and Schlesinger, 1995; Delgado-Baquerizo et al., 2013). Our results show a lower investment into AP under semi-dry to dry conditions providing support for such shifts in nutritional strategies on large spatial scales (e.g., Feng et al., 2016).

Soil pH was negatively correlated with modeled AP across Europe (Figure 6A), in agreement with the AP measurements based on acidic soils (Dick et al., 2000). Soils with high pHs are generally associated with fewer amino acid functional groups that are essential for catalysis (Dick et al., 2000). Clay content had a non-linear but weak relationship with modeled AP across Europe: increasing clay content increased AP in soils with low clay content (<10%) but decreased AP in soils with high clay content (>10%) (Figure 6E). Clay particles are characterized by many reactive binding sites for P and AP leading to the deactivation of the latter (Rao et al., 2000). Thus, other strategies than AP, like extraction of chelating agents and acids, might be advantageous in clay rich soils.

#### Relationships Between Modeled AP and Net Primary Production

The relationships between abiotic (climate, soils) factors and AP generally are in qualitative agreement with the results from most experimental studies, which provided evidence of a positive association between AP and environmental resources and conditions (water, temperature, and nutrients). Our analysis showed that MODIS-NPP, which covaries with climate, is also statistically associated with modeled AP (Figure 6K). Generally, our results are consistent with the mechanism that adequate resources (temperature, water and nutrients) can sustain a higher production and P demand for plants and microbes, thus larger AP activity to increase mineralization rates (Margalef et al., 2017). However, the co-relationship between AP and NPP is weaker than that with climatic variables and TN (Figures 6G–I,K), which indicating that the AP pattern is not only determined by P demand by biota but also by climate and soil nutrients.

#### Relationships Between Modeled AP and Vegetation and Soil Types

Modeled AP differed among the soil types, with higher activities in Oxisols and Ultisols (Figure S8) in line with earlier findings (Acosta-Martinez et al., 2007). These soils are commonly poor in P and thus a high recycling efficiency of organic P is an adaption to these conditions (Vance et al., 2003). We found that AP was higher in evergreen broadleaf forest than deciduous forests (Figure S8), consistent with the finding by Redel et al. (2008) of higher litter accumulation and lower P cycling in deciduous forests. Margalef et al. (2017) found that soil phosphatase was significantly higher in angiosperm than gymnosperm forests, likely because gymnosperm tend to grow on soils which have a lower biological activity as an adaptation to harsh conditions and thus have the associated disadvantage of low N-use efficiency.

### Implication and Uncertainties

The BPN models successfully extrapolated the site AP measurements on the Europe region. However, there are some inevitable uncertainties due to the gap-filling of input predictors which were not reported in the original literature. The extracted values from gridded datasets are biased to some extent, e.g., due to the coarse spatial resolution of predictor maps as some variables like TN, TP, and Clay vary strongly within very short (cm-m) distance (Lechowicz and Bell, 1991; Iqbal et al., 2005). As a consequence, the covariation between observed and extracted values of TN, TP, and Clay are not very strong (Figure S1).

However, non-linear BPN models are considered to be more sensitive to the orders of predictor values rather than linear variation among predictor values. This advantage of BPN compared to linear models reduces the influence of biases in gap-filling on AP predictions. The spearman rank correlations between observed and extracted values for Europe sites are found to be significant for all variables except for Clay (Figure S1). Thus, the data quality seems in general sufficient for the BPN model. Still, the BPN model might underestimate the driving effect of Clay on AP pattern due to the large biases. High-quality gridded datasets especially for soil clay content are required, to reduce the uncertainty in the extrapolations based on machine learning methods.

## Conclusions

This study is the first quantification of AP on continental scale by extrapolating the site observations of AP to a spatial explicit map of AP which can be used to benchmark land surface models for temperate regions. This study also provided the comprehensive overview of the factors driving the spatial variations of phosphatase activity on continental scale. The extent to which plants can upregulate P recycling under future climate changes could be limited by climatic and soil factors which are currently not accounted for in global models (e.g., Goll et al., 2012). Further, we identified the need for more measurements in tropical, arid, boreal, or alpine ecosystems, so as to be able to extend our approach to global scale.

## Data Availability Statement

The datasets of predictors used in this study are publicly available and can be accessed by the links we provided in Supporting Information. The code for BPN and RT can be accessed on Github: https://github.com/yansun8/AP_extropolation. Other data are available upon request from the authors.

## Author Contributions

YS, DG, and SP designed the research. YS and DG wrote the paper. YS did the extrapolation of AP and analyzed the data. OM and DA collected the dataset of site measurements. PC, JS, and JP helped revise this paper.

### Conflict of Interest

The authors declare that the research was conducted in the absence of any commercial or financial relationships that could be construed as a potential conflict of interest.
