# Supplementary material for: Spatial Pattern and Environmental Drivers of Acid Phosphatase Activity in Europe
Source: Front Big Data. 2020 Jan 23;2:51. doi: 10.3389/fdata.2019.00051 (PMC7931918; doi:10.3389/fdata.2019.00051)
Supplement: Supplementary file 1 [file Presentation_1.pdf]

Supplementary Material

S1. Datasets sources for 12 predictors used in this study

We used the best fitted data-driven metrics for data gap-filling for input predictors and AP extrapolation for whole Europe region based on the comparison of rank correlation (ρ) between observed and extracted values (Table 1; Figrue S1). SoilpH, TN, TP and Clay are gathered by using Land Use and Coverage Area Frame Survey (LUCAS) topsoil (0~20cm) database with spatial resolution of 500m, which is one of the world's largest and most comprehensive, harmonized continental-scale soil database generated by using hybrid approaches (Ballabio et al., 2016, 2019; https://esdac.jrc.ec.europa.eu/resource-type/datasets). OC is gathered by using Soilgrids data with spatial resolution of 250m which contains predictions of OC at seven standard depths: 0 cm, 5 cm, 15 cm, 30 cm, 60 cm, 100 cm and 200 cm (Hengl et al., 2017; https://files.isric.org/soilgrids/data/recent/). We first calculated the weighted average of OC (wt %) within 0~15 cm soil depth as below, according to Hengl et al. (2017):

$$OC = \frac{1}{15} \cdot \frac{1}{2} \sum_{k=1}^{N-1} (D_{k+1} - D_k)(OC_k + OC_{k+1})$$

where N is the number of depths, D_k is the k-th depth and OC_k is the OC at depth D_k.

Climatic information (MAT, AMP and MAP) was obtained from WorldClim datasets (version 2.0; http://worldclim.org/version2), which was produced by interpolation from weather stations combined with satellite datasets (Fick & Hijmans, 2017). We used average NPP across 2000-2013 from MODIS (MOD17A3) (Running et al., 2004; Zhao et al., 2005; Turner et al., 2006; http://files.ntsg.umt.edu/data/NTSG_Products/MOD17/MOD17A3). SoilType was extracted from global map of soil suborders derived by World Soil Information (ISRIC; http://www.isric.org/content/faq-soilgrids). VegType was obtained from the MODIS Land Cover Type product (MOD12Q2; Friedl & Sulla-Menashe, 2015; https://lpdaac.usgs.gov/dataset_discovery/modis/modis_products_table/mcd12q2). Since there is no available Po dataset, we estimated the Po from global map of soil labile inorganic phosphorus (labile Pi; half degree; Yang et al., 2013), the only available global datasets for the content of soil P forms,

multiplied by ratio of the labile Pi and labile Po. The ratios for each soil suborder are derived from collection of soil P measurements at depth of 50 cm by Yang et al. (2013) (Sun et al., 2017).

For the extrapolation of AP map for Europe on 10km, we used gridded datasets above which was resampled into 10km. For the extrapolation of AP map for global region exclude Europe region on 10km, we used global gridded datasets from ISRIC-WISE Global Dataset of Derived Soil Properties for TN and Clay (Batjes et al., 2015; https://data.isric.org/geonetwork/srv/chi/catalog.search#/metadata/dc7b283a-8f19-45e1-aaed-e9bd515119bc), and used Global Soil Dataset for Earth System Modelling (GSDE) for TP (Shangguan et al., 2014; http://globalchange.bnu.edu.cn/research/soilw). We used the same gridded datasets with that for Europe for other predictors (USDA-soiltype, MODIS-NPP and WorldClim data, see above) (Table S1). Both of those datasets have a spatial resolution of 1km and was resample into 10km before the extrapolation.

S2. Gap filling and pre-processes of observation data sets and predictors

We used total C content instead of sites with soil pH <6 for sites with no OC data, because inorganic C is largely in carbonate forms that do not occur in acid soils (Nelson & Sommers, 1996). The predictors SoilpH, OC, TN, TP and Clay were reported together with AP in the original publications takes proportion of 95%, 28%, 92%, 25% and 23% of 126 European sites *(Data set A*). In other cases, these variables were obtained from gridded databases with different spatial resolutions from 250m~1 km based on geographical coordinates of each measurement site (LUCAS; Ballabio et al., 2016, 2019). Climatic variables (MAT, MAP and AMP) (WorldClim; Fick & Hijmans, 2017), NPP (MOD17), SoilType (ISRIC; Hengl et al., 2017) and VegType (MOD12Q2) were extracted from global databases by using the coordinates of the measurement site.

Environmental data often have skewed distributions, such as soil nutrient concentrations (Blackwood, 1992). Fitting lognormal distributions is commonly used to represent this kind of data in statistical analysis (Blackwood, 1992). We tested the distribution of 296 measurements of AP and of the 10 numerical predictors for all global pixels (Figure S2). The acid phosphatase activity was found to follow a lognormal distribution, so that AP was log-transformed. The content of soil nutrients (OC, TN, TP and Po) are also log-normally distributed. Additionally, NPP, AMP and MAP also follow lognormal distribution. Therefore, all these predictors were also log-transformed.

S3. Optimal number of neurons for BPN

The performances of a BPN is partly determined by the number of neurons in each hidden layer. To decide the optimal number of neurons, we used exhaustive combinations of 1-20 neurons for two hidden layers to evaluate the performance of BPN models with respect to number of neurons. The BPN models were trained 1000 times using different training-testing subsets (85% for training datasets, 15% for test datasets). We found that the lowest root mean square error (RMSE) for test datasets occurs in BPN framework with about 10 neurons for hidden layer 1 and 5 neurons for hidden layer 2 (Figure S3)

S4. Detection of outliers

There is a very low accuracy of 19% of explained variance of AP over all measurements, accompanied by a very high root mean square error (RMSE) of 18.2 μmol g^{-1} h^{-1} (Table 2). 9 outliers (*Site A-I*) with extreme bias (absolute bias >20 μmol g^{-1} h^{-1} and the relative bias >50%, Figure 3) were detected. Those seven outliers are located in central England (*Site A*), Eastern Spain (*Site C, H, I*), Poland (*Site D*), and Western Spain (*Site B, E, F, G*). Those extreme biases are attributed to that BPN is insufficient to reproduce the AP observations for particular sites of which predictors falling in the edge of their distributions. For example, *Site A* is out of the margin of Clay. *Site C* is out of the the margin of Clay, OC and TN. The information of the marginal predictors for each outlier are listed in Table S3.

S5. Compare the performance of the BPN on tropical sites and temperate sites outside Europe

To access if the AP extrapolation for global region is acceptable, we compared the performance of the BPN on tropical sites and temperate sites outside Europe. The predicted AP were generated (1) by reproducing the AP for temperate and tropical sites with complete information using and (2) by extracting from the global pattern of predicted AP according to the coordinates of the measurement sites (Figure S12). Note that TN was detected to be largely affect AP pattern (Figure 8) but have a low accuracy for extracted TN from global gridded dataset ISRIC-WISE compared with the values reported by literature (Figure S1), we only reproduced the sites with original TN for (1).

S6. Understanding the failure to extrapolate AP to tropical region

The relationships between AP and environmental factors reported for tropical regions differed greatly from our model for Europe, which could account for the failure to extrapolate the BPN model trained using European sites to tropical regions. Climates (precipitation and temperature) in tropical rainforest are generally not factors limiting growth or more homogenous than in extra-tropics. Soil

conditions instead of climate are thus considered to be the most important factors in driving biological activity and thus AP.

Highly weathered tropical soil in particular always has large amounts of highly reactive minerals (Vitousek et al., 2010) that can interact with and deactivate AP. Because these enzymes interact with these minerals (Dick & Tabatabai, 1987) and can potentially lead to differences between AP from microbes and plants (Kitayama, 2013) that are absent in Europe. Gridded datasets of soil variables, such as Po, are associated with large uncertainties, which strongly limits BPN extrapolation for tropical regions. Besides, soil environment (pH, nutrient contents, texture) can also vary within short distances. The representativeness of samples will thus strongly influence model calibration and may lead to large uncertainties in AP estimates, which cannot be solved by our BPN model. More measurements of AP in tropical regions are needed to help us understand the physiological basis of P-use strategies in tropical forest ecosystems and reduce the uncertainty in estimates of tropical AP estimates.

References

Blackwood, L. G. (1992). The lognormal distribution, environmental data, and radiological monitoring. *Environmental monitoring and assessment*, *21*(3), 193-210.

Nelson, D. W., & Sommers, L. E. (1996). Total carbon, organic carbon, and organic matter. *Methods of soil analysis part 3—chemical methods, (methodsofsoilan3)*, 961-1010.

Table S1 Predictors used in extrapolation of AP for regions outside of Europe.

Predictor Name	Abbreviation	Source	References
Soil organic carbon	OC	Soilgrids	Hengl et al., 2017
Soil pH	SoilpH		
Soil total nitrogen	TN	ISRIC-WISE	Batjes et al., 2015
Soil clay content	Clay		
Soil total phosphorus	TP	GSDE	Shangguan et al., 2014
Soil labile organic phosphorus	Po	Global maps of the soil P contents for different P forms and<br><br>USDA soil types and ratio of labile inorganic P and labile organic P	Yang et al., 2013;<br><br>Hengl et al., 2017;<br><br>Sun et al., 2017
Net primary productivity	NPP	MODIS-NPP (MOD17A3; mean value during 2000-2014)	Running et al., 2004; Zhao et al., 2005; Turner et al., 2006
Mean annual temperature	MAT	WorldClim	Fick and Hijmans, 2017
Yearly temperature amplitude	AMP		
Mean annual precipitation	MAP		
Soil type (categorical variable)	SoilType	Soilgrids & USDA class	Hengl et al., 2017
Vegetation type (categorical variable)	VegType	MODIS	Friedl et al, 2015

Table S2 Regression statistics for Observed vs Predicted acid phosphatase activity derived from (BPN) and (RT) models. Statistics that excluding outliers were also provided. Numbers in parenthesis indicate the number of outliers.

	R^2		RMSE (μmol g^{-1} h^{-1})	
	BPN	RT	BPN	RT
With outliers	0.192	0.096	18.2	19.7
Without outliers	0.578 (9)	0.487 (13)	6.83	7.85

Table S2 Information of the marginal predictors for 9 outliers detected by BPN.

Sites	Predictors falling in the edge of their distributions
A	Clay
B	NPP, Soiltype
C	Clay, pH, TP, OC, TN
D	Labile Po, NPP, OC, TN, Soiltype
E	NPP
F	TP, labile Po
G	NPP
H	TN
I	TP

Figure S1 The comparison of Pearson correlation and rank correlation (ρ) between observed predictors and extracted values from 4 different gridded metrics.

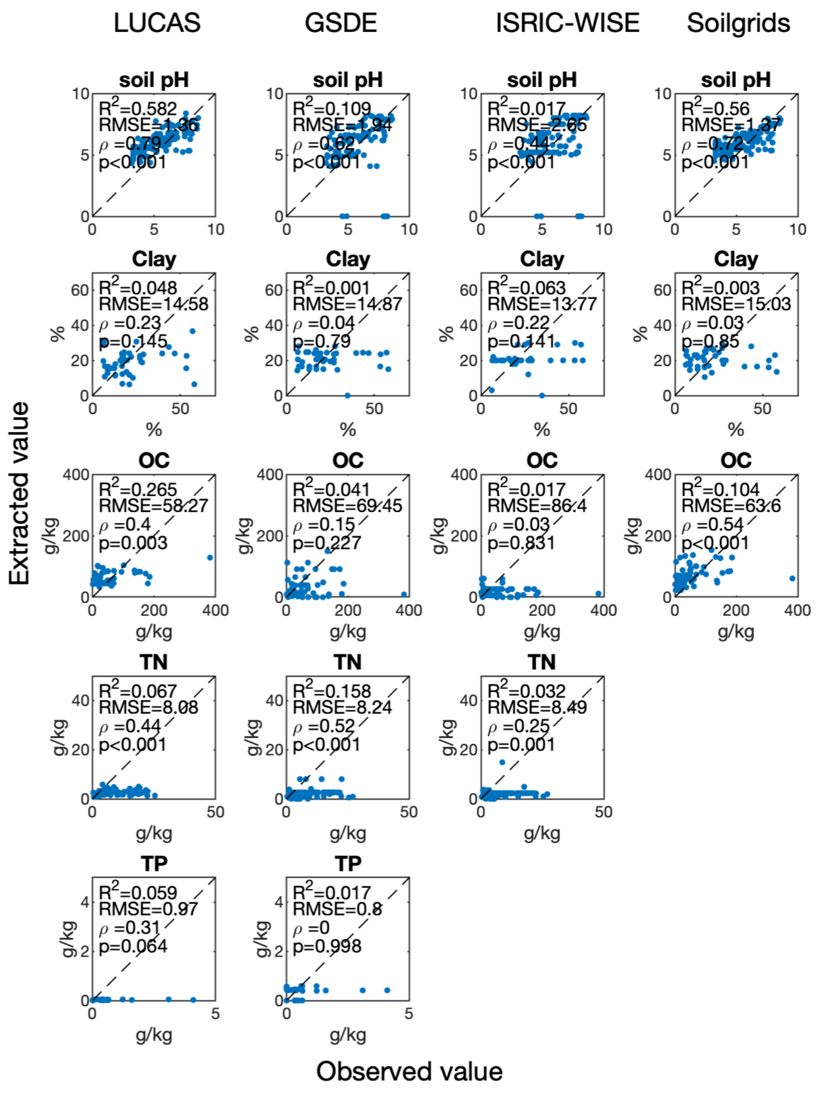

Figure S2 The distribution of 10 numerical predictors used in this analysis for global pixels.

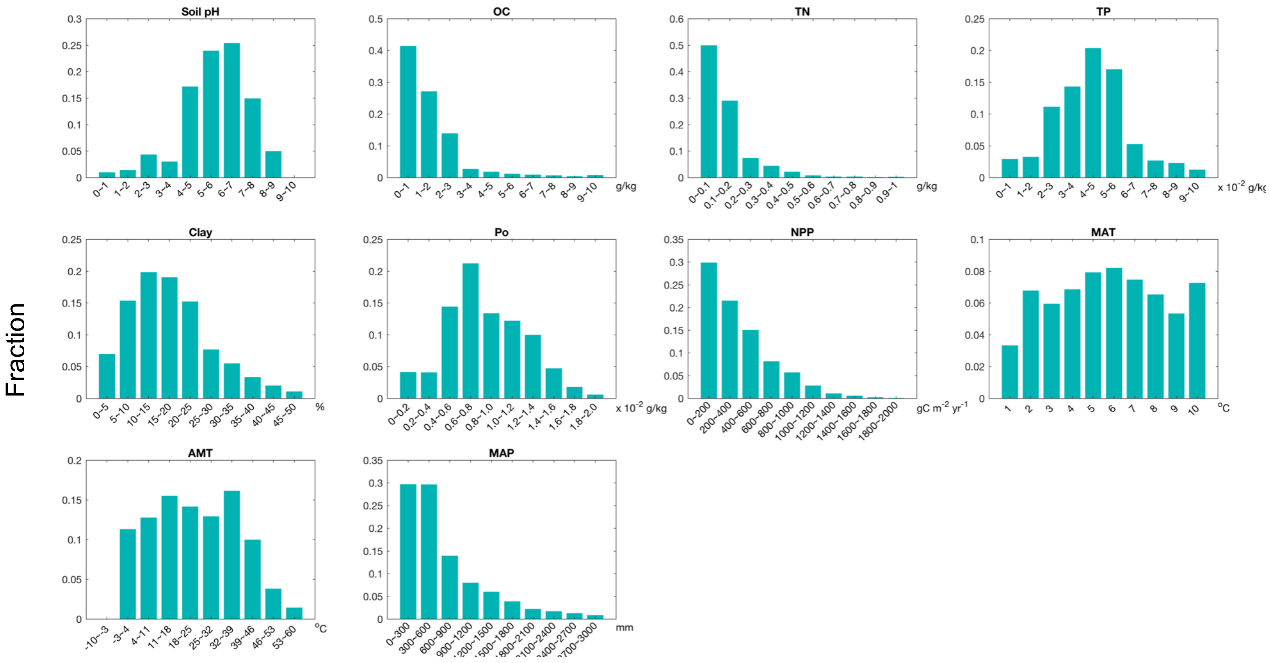

Figure S3 The root mean square error (RMSE; μmol g^{-1} h^{-1}) on train and test datasets by using different number of neurons in two hidden layers for back-propagation models.

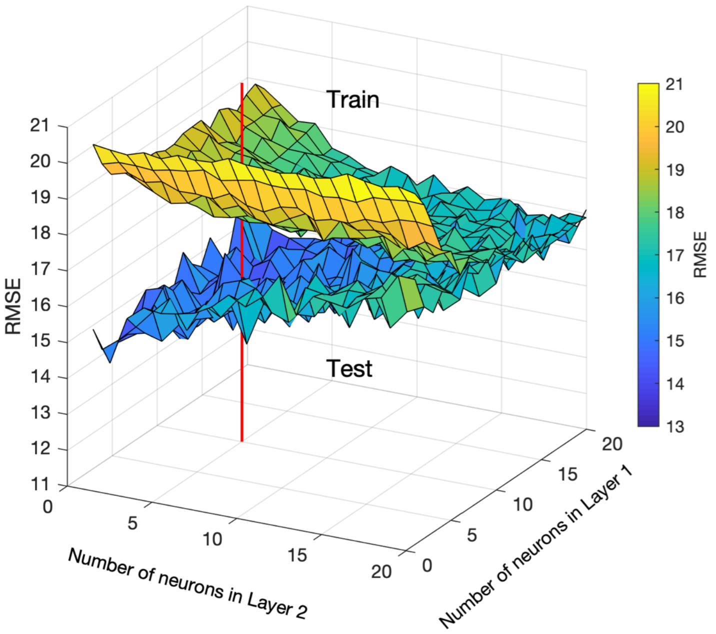

Figure S4 The distribution of 9 sites with very biased estimates of AP by BPN.

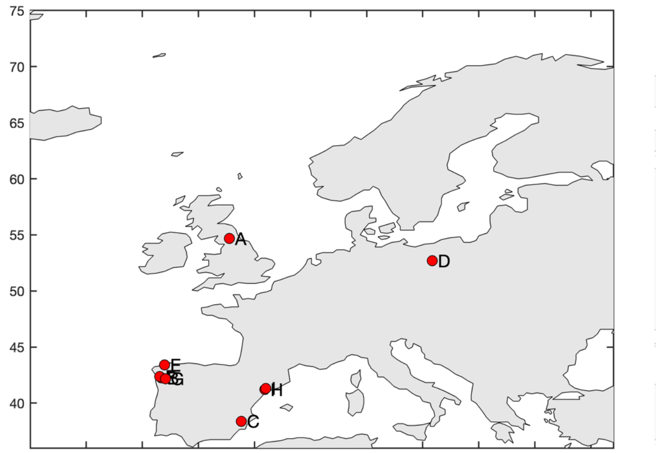

Figure S5 The distribution of 12 predictors for all of 126 European sites. 8 soil types and 11 biome types are considered: Evergreen Needleleaf Forest (ENF), Evergreen Broadleaf Forest (EBF), Deciduous Broadleaf Forest (DBF), Mixed forest (MF), Open Shrublands (OS), Woody Savannas (WS), Savannas (S), Grasslands (GR). 9 sites with very biased estimates of AP are highlighted by grey dots and alphabet corresponding to Figure S4.

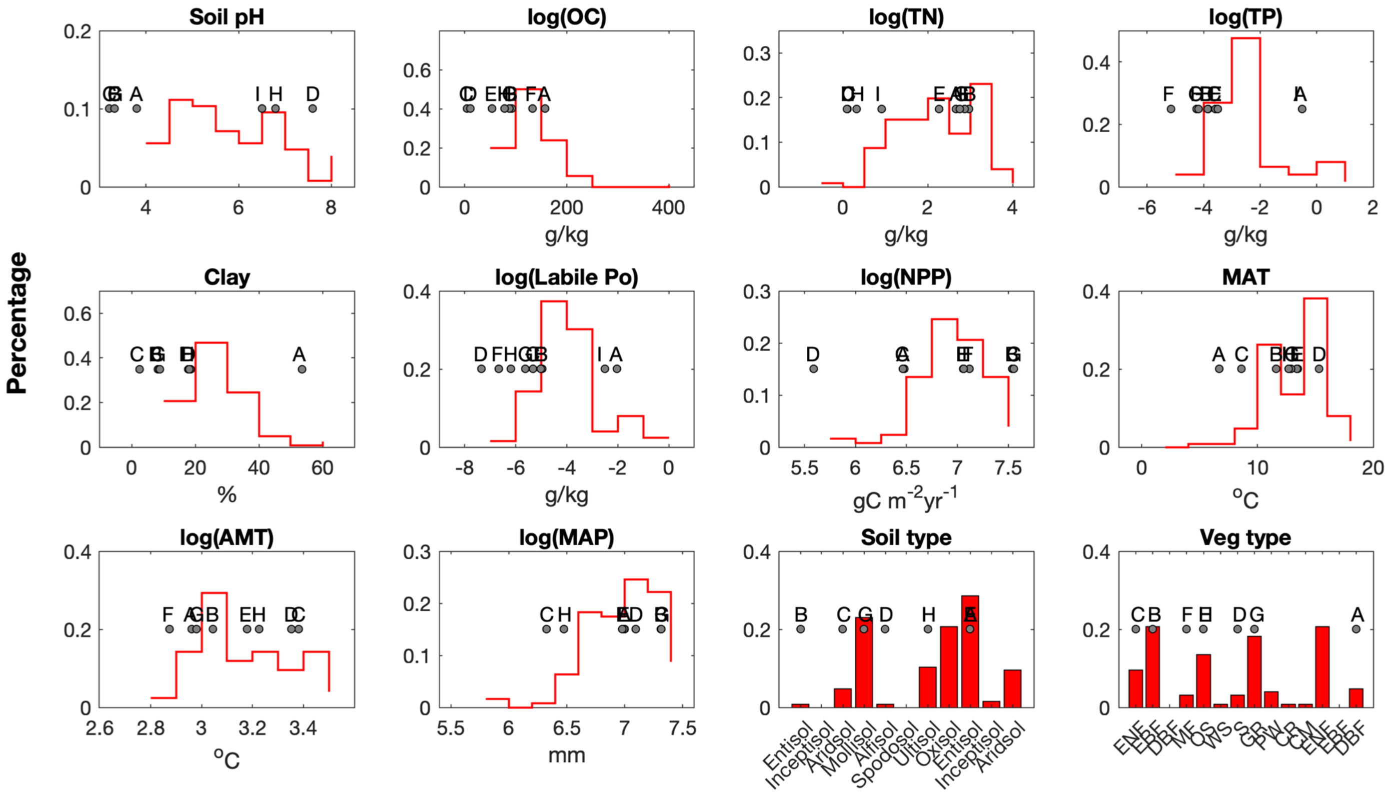

Figure S6 Performance of Regression Tree models (RT) on 126 European sites following hold-one-out framework. (A) shows the Predicted vs Observed acid phosphatase activity (AP) for all sites, while (B) shows the Predicted vs Observed AP with excluding 7 outliers (grey circles). Dashed lines indicate the 1:1 line. Blue lines refer to the regression line between Predicted vs Observed AP. Blue and grey error bars show the 25%~75% quantiles of predicted AP, while orange error bars show the 10%~90% quantiles of predicted AP. (C) shows the distribution of 13 outliers with very biased estimates of AP by RT.

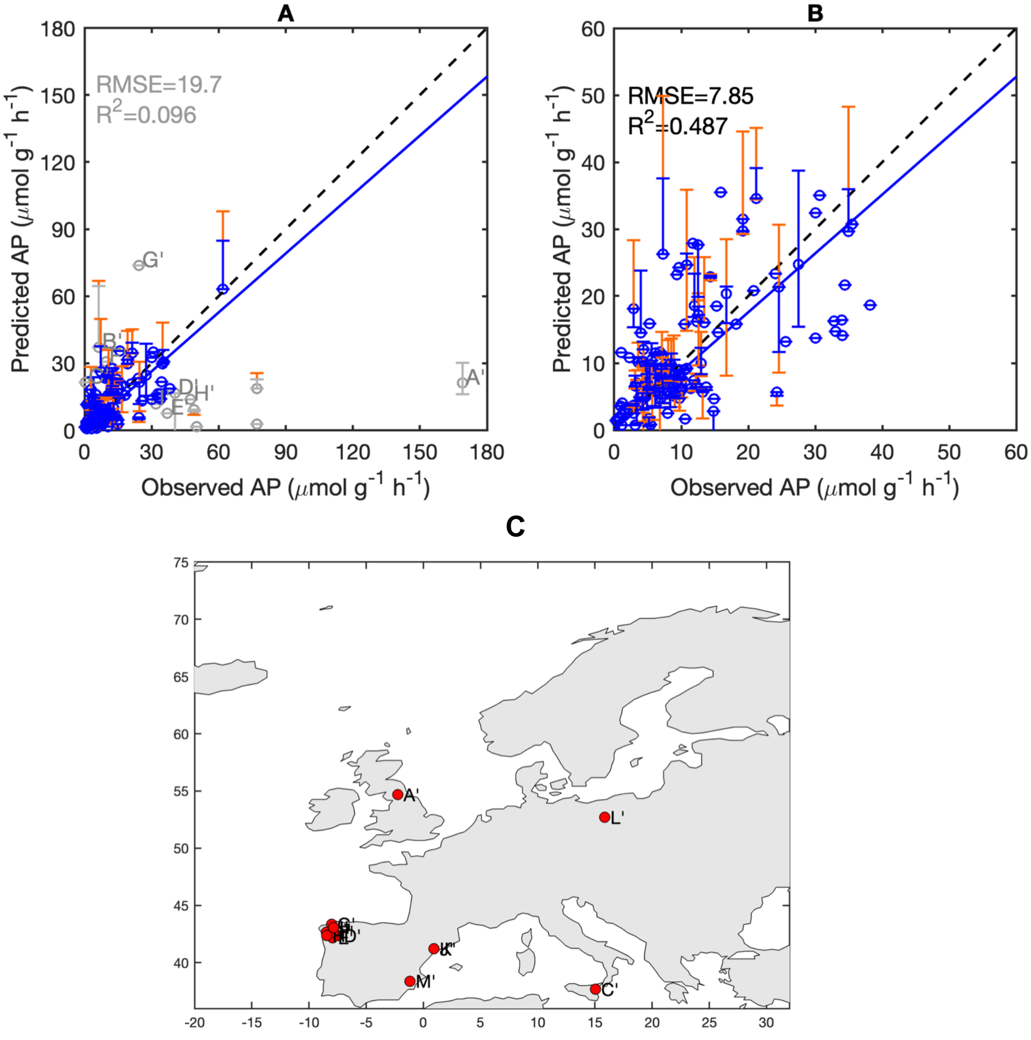

Figure S7 The extrapolated spatial pattern of 25% (A) and 75% (B) quantile estimates of acid phosphatase activity by BPN on Europe.

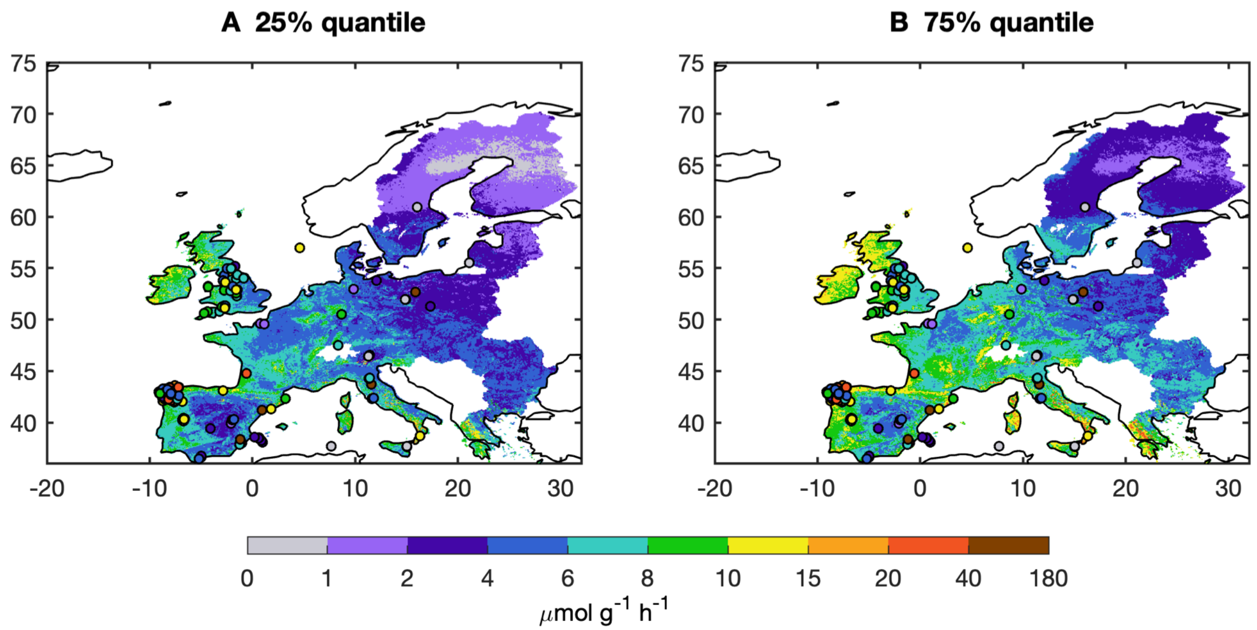

Figure S8 Median estimates of AP for different 8 soil types and 11 biome types: Evergreen Needleleaf Forest (ENF), Evergreen Broadleaf Forest (EBF), Deciduous Broadleaf Forest (DBF), Mixed forest (MF), Open Shrublands (OS), Woody Savannas (WS), Savannas (S), Grasslands (GR). Error bars show the 25%~75% quantiles of AP for same soil (or biome) type.

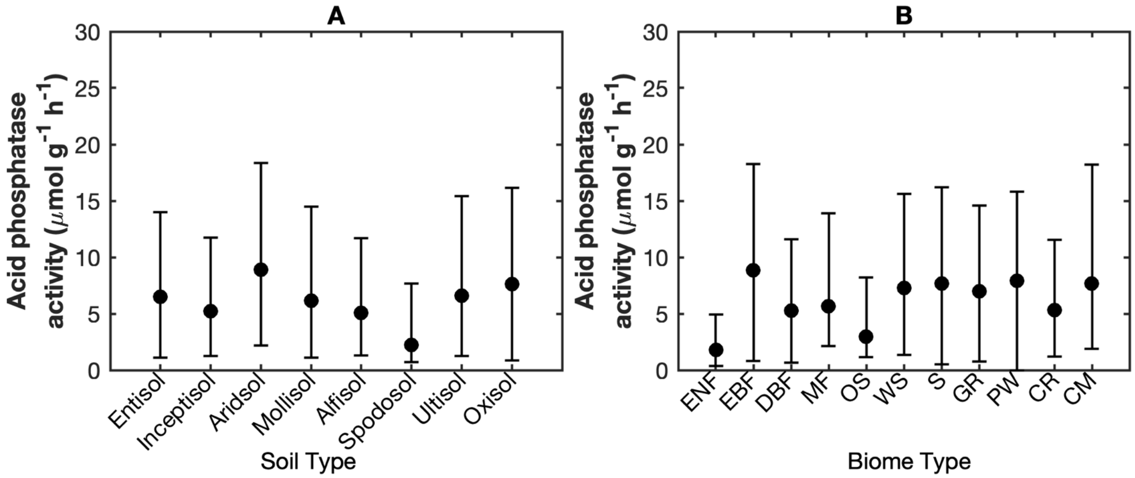

Figure S9 Explanation of climates (MAT, AMT, MAP) to NPP for Europe region. The regression analysis was conducted by using a spatial moving window of 4.5° × 4.5°, of which climates and NPP were firstly resample into half degree by using area-weighted mean methods.

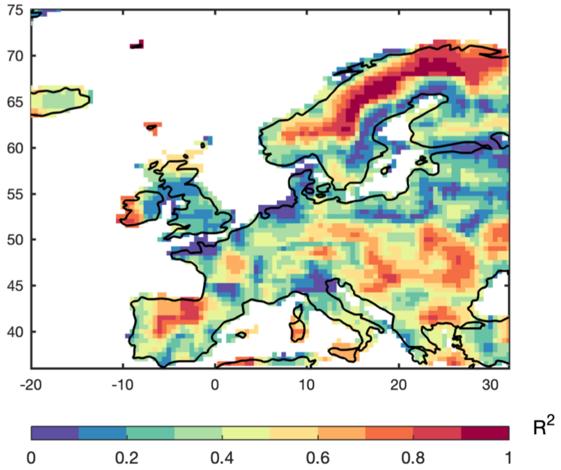

Figure S10 The distribution of 12 predictors for all of 126 European sites (*Data set A*; black) and 87 temperate sites outsides Europe (*Data set C*; blue) and 54 tropical sites (*Data set D*; red). 8 soil types and 11 biome types are considered: Evergreen Needleleaf Forest (ENF), Evergreen Broadleaf Forest (EBF), Deciduous Broadleaf Forest (DBF), Mixed forest (MF), Open Shrublands (OS), Woody Savannas (WS), Savannas (S), Grasslands (GR).

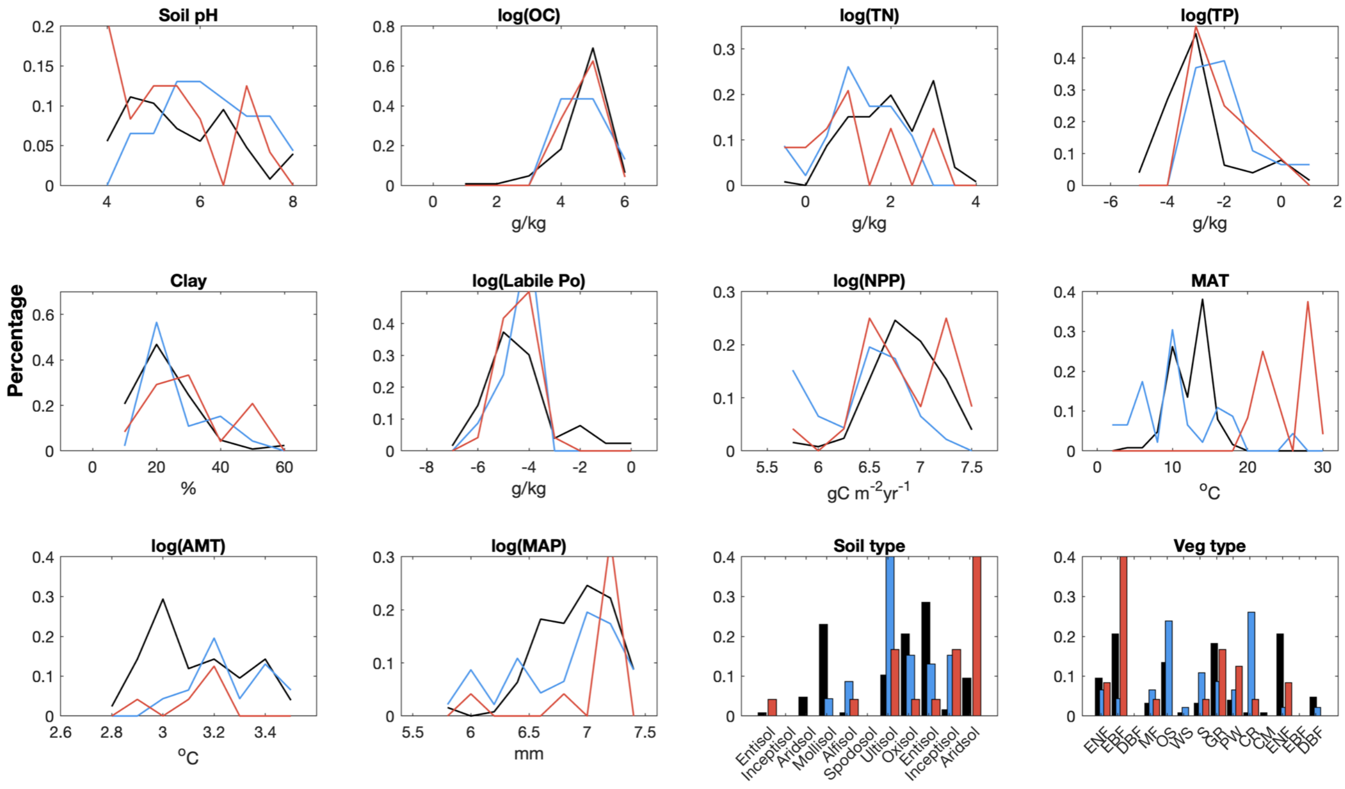

Figure S11 The relationship between partial correlation coefficients of AP to TP (blue) and labile Po (red) and aridity index (P/PET). The aridity index is calculated as the ratio of mean annual precipitation and mean potential evapotranspiration across 1981-2018 that derived from Climate Research Unit (CRU) datasets (CRU TSv4.03). Blue and red lines indicate the regression between partial correlation coefficients and aridity index. Grey shading indicates where the partial correlation relationship is not significant (*p*>0.1).

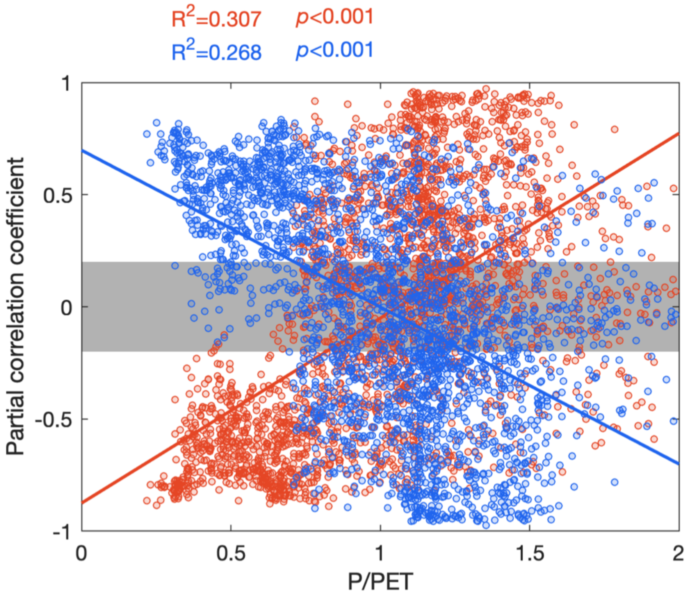

Figure S12 Performance of the back-propagation networks (BPN) on temperate sites (excluding Europe) (A, C, E, G) and tropical sites (B, D, F, H). A and B show the comparisons between observed acid phosphatase activity (AP) and AP extracted from the extrapolated global pattern of AP (Figure 5). C-H show the comparisons between observed AP and predicted AP by using BPN models and predictor information on site scale. C and D show the performance of BPN models on all sites (*Data set C* and *D*). E and F exclude the sites with gap-filled soil total nitrogen (TN), while G and H exclude the sites with mean annual temperature (MAT) <5°C and MAT>20°C. The solid lines

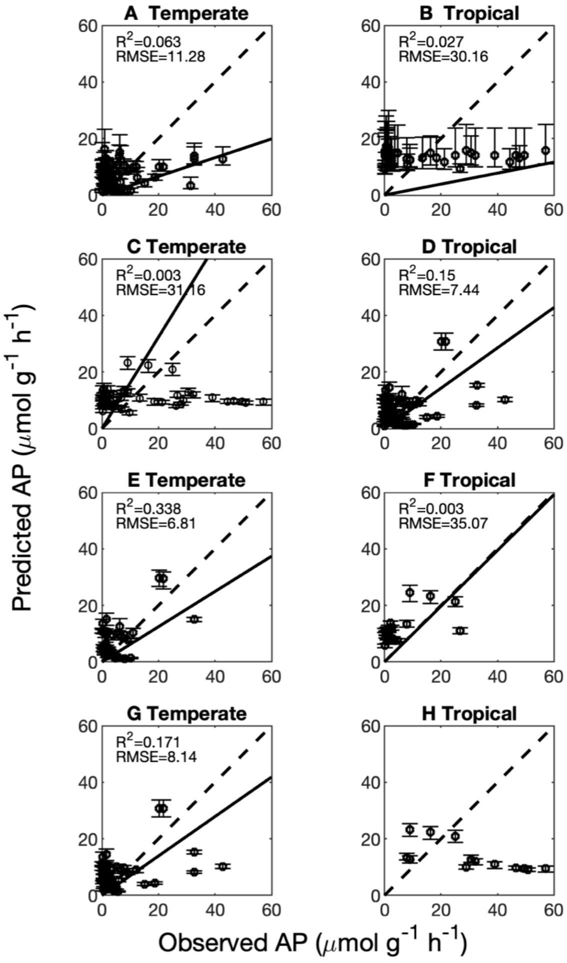

indicate the regression lines between predicted vs observed AP. The dashed lines indicate the 1:1 lines. The error bars indicate the 10% and 90% quantiles of predicted AP.
